# *TERT* promoter mutations contribute to *IDH* mutations in predicting differential responses to adjuvant therapies in WHO grade II and III diffuse gliomas

**DOI:** 10.18632/oncotarget.4549

**Published:** 2015-07-09

**Authors:** Zhen-Yu Zhang, Aden Ka-Yin Chan, Xiao-Jie Ding, Zhi-Yong Qin, Christopher S. Hong, Ling-Chao Chen, Xin Zhang, Fang-Ping Zhao, Yin Wang, Yang Wang, Liang-Fu Zhou, Zhengping Zhuang, Ho-Keung Ng, Hai Yan, Yu Yao, Ying Mao

**Affiliations:** ^1^ Department of Neurosurgery, Huashan Hospital, Fudan University, Shanghai, China; ^2^ Department of Anatomical and Cellular Pathology, Prince of Wales Hospital, The Chinese University of Hong Kong, Shatin, N.T., Hong Kong, China; ^3^ Surgical Neurology Branch, National Institute of Neurological Disorders and Stroke, National Institutes of Health, Bethesda, Maryland, USA; ^4^ Genetron Health, Inc., Chaoyang District, Beijing, China; ^5^ Department of Neuropathology, Huashan Hospital, Fudan University, Shanghai, China; ^6^ Department of Pathology, Duke University Medical Center, The Preston Robert Tisch Brain Tumor Center, The Pediatric Brain Tumor Foundation Institute, Durham, North Carolina, USA

**Keywords:** *TERT* promoter, *IDH*, gliomas, radiation therapy, chemotherapy

## Abstract

*IDH* mutations frequently occur in WHO grade II and III diffuse gliomas and have favorable prognosis compared to wild-type tumors. However, whether *IDH* mutations in WHO grade II and II diffuse gliomas predict enhanced sensitivity to adjuvant radiation (RT) or chemotherapy (CHT) is still being debated. Recent studies have identified recurrent mutations in the promoter region of *telomerase reverse transcriptase* (*TERT*) in gliomas. We previously demonstrated that *TERT* promoter mutations may be promising biomarkers in glioma survival prognostication when combined with *IDH* mutations. This study analyzed *IDH* and *TERT* promoter mutations in 295 WHO grade II and III diffuse gliomas treated with or without adjuvant therapies to explore their impact on the sensitivity of tumors to genotoxic therapies. *IDH* mutations were found in 216 (73.2%) patients and *TERT* promoter mutations were found in 112 (38%) patients. In multivariate analysis, *IDH* mutations (*p* < 0.001) were independent prognostic factors for PFS and OS in patients receiving genotoxic therapies while *TERT* promoter mutations were not. In univariate analysis, *IDH* and *TERT* promoter mutations were not significant prognostic factors in patients who did not receive genotoxic therapies. Adjuvant RT and CHT were factors independently impacting PFS (RT *p* = 0.001, CHT *p* = 0.026) in *IDH* mutated WHO grade II and III diffuse gliomas but not in *IDH* wild-type group. Univariate and multivariate analyses demonstrated *TERT* promoter mutations further stratified *IDH* wild-type WHO grade II and III diffuse gliomas into two subgroups with different responses to genotoxic therapies. Adjuvant RT and CHT were significant parameters influencing PFS in the *IDH* wt/*TERT* mut subgroup (RT *p* = 0.015, CHT *p* = 0.015) but not in the *IDH* wt/*TERT* wt subgroup. Our data demonstrated that *IDH* mutated WHO grade II and III diffuse gliomas had better PFS and OS than their *IDH* wild-type counterparts when genotoxic therapies were administered after surgery. Importantly, we also found that *TERT* promoter mutations further stratify *IDH* wild-type WHO grade II and III diffuse gliomas into two subgroups with different responses to adjuvant therapies. Taken together, *TERT* promoter mutations may predict enhanced sensitivity to genotoxic therapies in *IDH* wild-type WHO grade II and III diffuse gliomas and may justify intensified treatment in this subgroup.

## INTRODUCTION

Diffuse gliomas are the most common primary malignant brain tumors with the propensity to infiltrate adjacent brain parenchyma [1]. According to The World Health Organization (WHO), based on histological criteria, diffuse gliomas are categorized into astrocytoma, oligodendroglioma and oligoastrocytoma, and graded from grade II to IV [2]. Among astrocytic glioma, also known as glioblastoma multiforme (GBM), patients with grade IV tumors have relatively better but variable survivals than patients with grade II and III tumors. Due to their variable prognosis and difficulties in designing and evaluating clinical trials in WHO grade II and III diffuse gliomas, treatment strategies on these gliomas are still controversial [3–5].

Recently, molecular biomarkers have become important in the classification of WHO grade II and III diffuse gliomas and prediction of survival and response to treatment. Chromosome 1p/19q codeletion has been associated with favorable clinical outcome and enhanced chemoradiosensitivity in oligodendroglial tumors [6–8]. Mutations in *isocitrate dehydrogenase 1* (*IDH1*) and *IDH2* have been discovered in the majority of WHO grade II and III diffuse gliomas and secondary GBM [9–11]. Furthermore, there is strong evidence that patients with *IDH* mutated gliomas across all tumor grades exhibit better overall survivals compared to their wild-type counterparts [11–15]. Far less certain is whether this survival benefit can be explained by improved response to adjuvant genotoxic therapies like radiation therapy (RT) or chemotherapy (CHT) or is attributable to differences in intrinsic tumor behavior. While some prospective and retrospective studies have demonstrated greater response rates to adjuvant therapies and longer progression-free survival (PFS) in the *IDH* mutated subset of WHO grade II and III diffuse gliomas [14, 15], others have failed to observe the same findings [12, 13].

Frequent mutations in the promoter region of *telomerase reverse transcriptase* (*TERT*) are detected in various types of tumors, including gliomas [16–18]. The *TERT* gene encodes the catalytic subunit of telomerase, an enzyme that elongates telomeres in cells, and prevents chromosomal degradation from multiple rounds of mitosis [19, 20]. Somatic *TERT* promoter mutations, most commonly being C228T and C250T, generate a new binding site (5′-TTCC-3′) for E-twenty-six (ETS) transcription factors, which increases *TERT* gene transcription and indicates that *TERT* mutations contribute to tumorigenesis via telomerase activation [16, 17, 20, 21]. In glioma genomics, *TERT* promoter mutations are frequently found in over 70% of primary GBM and oligodendrogliomas, and less frequently in oligoastrocytomas and WHO grade II and III diffuse astrocytomas [18, 19, 21]. Furthermore, we and others showed that *TERT* promoter mutations in combination with *IDH* mutation, are promising prognostic indicators of survival in glioma [17, 19, 20, 22]. The role of *TERT* promoter mutations in predicting responses to adjuvant genotoxic therapies in gliomas remains relatively unexplored. In this study, we performed mutational analysis for *TERT* promoter and *IDH* in a large series of WHO grade II and III diffuse gliomas and summarized the patient outcome in response to adjuvant therapies.

## RESULTS

### Clinical and pathological characteristics of the cohort

Out of 295 total patients, there were 179 males and 116 females in the series with a male to female ratio of 1.54:1. The mean age at diagnosis was 42.6 ± 12.1 years. The mean duration of follow-up was 9.6 years. All patients underwent tumor resection: 153 patients had total resection, 103 patients had subtotal resection, and the extent of resection in the remaining 39 cases could not be retrieved or evaluated based on available data. 231 patients (78.3%) received postoperative RT and 180 patients (61%) received CHT. In total, 246 patients (83.4%) were treated with some form of RT and/or CHT after surgery, while 49 (16.6%) patients received neither RT nor CHT. In the 231 patients receiving postoperative RT, radiation doses and fractions were available in 174 cases (75.3%). The radiation doses ranged from 52.0 Gy to 66.4 Gy with a mean dose of 59.1 Gy. In the 180 cases receiving postoperative CHT, chemotherapy strategies were available in 141 cases (78.3%). The chemotherapy protocols administered included temozolomide (TMZ, 43.3%) and alkylating agents such as semustine (MeCCNU, 39.0%), fotemustine (FCNU, 12.1%) and nimustine (ACNU, 5.7%).

*IDH* mutations were found in 216 (73.2%) cases while mutations in the *TERT* promoter were found in 112 (38%) cases. Among the 216 cases with *IDH* mutations, there were 206 cases harboring *IDH1* mutations and 10 cases harboring *IDH2* mutations. Among the 112 *TERT* promoter mutated tumors, C228T mutations were observed in 76 (67.9%) cases and C250T mutations were detected in 36 (32.1%) cases. Chromosome 1p/19q codeletion was detected in 73 (24.7%) WHO grade II and III diffuse gliomas. These data are shown in Table 1.

**Table 1:** Clinical, pathological, and treatment characteristics of the patient cohort (*n* = 295)

Factors	No. of cases	Percentage (%)
Sex		
Male	179	60.7
Female	116	39.3
Age		
Mean	42.6	
Standard deviation	12.1	
WHO grade		
Grade II	188	63.7
Grade III	107	36.3
Histology		
Astrocytic	178	60.3
Oligodendroglial/Oligoastrocytic	117	39.7
IDH mutation		
Mutant	216	73.2
Wild-type	79	30
TERT promoter mutation		
Mutant	112	38
Wild type	183	62
1p/19q codeletion		
Yes	73	24.7
No	222	75.3
Extent of resection*		
Complete	153	59.8
Incomplete	103	40.2
Primary RT		
Yes	231	78.3
No	64	21.7
Primary CHT		
Yes	180	61
No	115	39

* Extent of resection in 39 cases was unavailable.

### *IDH* mutations, not *TERT* promoter mutations, are independent prognostic factors in response to genotoxic therapies

**Figure 1:**
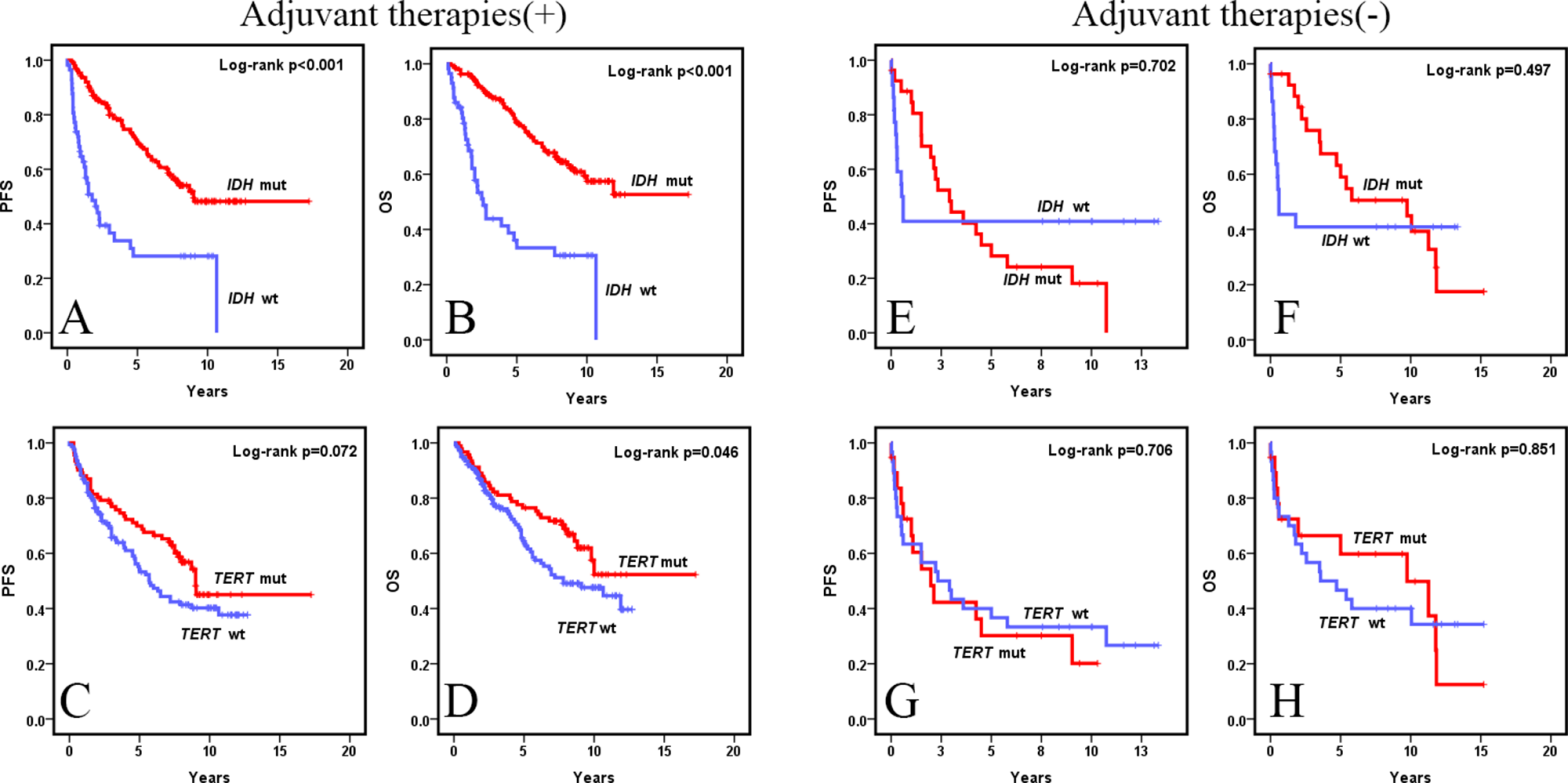
Kaplan-Meier survival curves (univariate analysis) of *IDH* and *TERT* promoter mutations for OS and PFS in WHO grade II and III diffuse gliomas with and without adjuvant therapies *IDH* mutations were associated with significantly longer OS **A.** and PFS **B.** in WHO grade II and III diffuse gliomas treated with genotoxic therapies after surgery but *TERT* promoter mutations were not significantly associated with longer PFS **C.** and were significantly associated with longer OS **D.** In the absence of genotoxic therapies after surgery, *IDH* mutations **E** and **F.** and *TERT* promoter mutations **G** and **H.** were not associated with significantly longer OS and PFS in WHO grade II and III diffuse gliomas.

We divided the entire series into two groups, based on postoperative therapies. Group A (*n* = 246) patients received adjuvant postoperative genotoxic therapies in the form of RT, CHT, or both and Group B (*n* = 49) patients had no additional treatment after surgery. Univariate analysis on Group A revealed patients with *IDH* mutated WHO grade II and III diffuse gliomas had significantly better PFS (*p* < 0.001) and OS (*p* < 0.001) than those with *IDH* wild-type WHO grade II and III diffuse gliomas (Fig. 1A-1B and Table S1). Multivariate analysis demonstrated *IDH* mutations (PFS *p* < 0.001, OS *p* < 0.001) and two other putative prognostic factors (WHO grade and extent of resection) to be independent predictors of PFS and OS in Group A (Table 2). However, the prognostic value of *IDH* mutation status was lost for PFS and OS in both univariate and multivariate analysis in Group B (Fig 1E-1F, Table 2, and Table S1). Both univariate and multivariate analyses revealed no prognostic significance for *TERT* promoter mutation status in both Group A and Group B (Fig. 1C-1D, 1G-1H, Table 2, and Table S1).

**Table 2:** Multivariate analysis of clinicopathological factors for PFS and OS in patients with WHO II and III diffuse gliomas who received adjuvant therapies (Group A, *n* = 246) and those who did not (Group B, *n* = 49) after surgery

Adjuvant therapies	Variables	PFS		OS	
		HR(95%CI)	*p*-value	HR(95%CI)	*p*-value
RT and/or CHT	Age	1.016(1.000–1.033)	0.054	1.020(1.002–1.039)	**0.031**
	WHO grade (Grade II vs III)	2.263(1.474–3.475)	**<0.001**	2.573(1.610–4.110)	**<0.001**
	Complete resection (Yes vs. No)	2.645(1.794–3.899)	**<0.001**	2.116(1.389–3.223)	**<0.001**
	*IDH* mutation (Yes vs. No)	2.424(1.524–3.854)	**<0.001**	2.652(1.632–4.308)	**<0.001**
	1p/19q codeletion (Yes vs. No)	1.736(0.996–3.027)	0.052	1.834(0.978–3.439)	0.059
	*TERT* promoter mutation(Yes vs. No)	1.043(0.659–1.650)	0.858	1.128(0.690–1.844)	0.632
No RT or CHT	Age	1.038(1.009–1.069)	**0.011**	1.037(1.004–1.070)	**0.026**
	WHO grade (Grade II vs III)	11.330(3.770–34.048)	**<0.001**	7.001(2.167–22.615)	**0.001**
	Complete resection (Yes vs. No)	1.528(0.665–3.508)	0.318	1.705(0.747–3.892)	0.205
	*IDH* mutation (Yes vs. No)	0.707(0.295–1.694)	0.436	0.751(0.314–1.795)	0.519
	1p/19q codeletion (Yes vs. No)	0.936(0.302–2.898)	0.908	1.738(0.510–5.923)	0.377
	*TERT* promoter mutation(Yes vs. No)	1.832(0.704–4.765)	0.215	1.665(0.635–4.366)	0.300

*p* values in bold were considered statistically significant

HR: hazard ratio; CI: confidence interval; RT: radiation therapy; CHT: chemotherapy.

### RT and CHT are clinical factors independently impacting the PFS in *IDH* mutated WHO grade II and III diffuse gliomas but not in *IDH* wild-type subgroup

**Figure 2:**
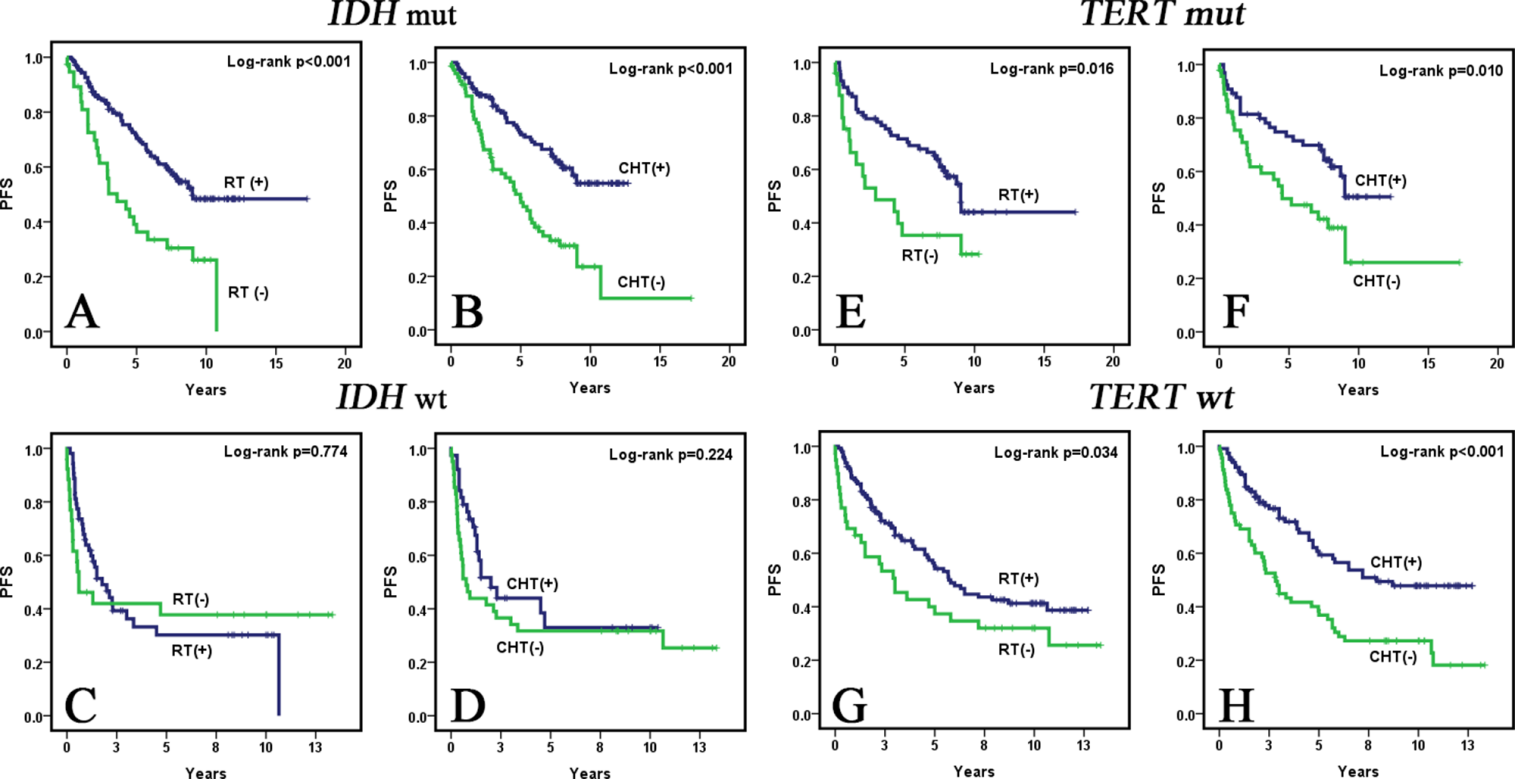
Kaplan-Meier survival curves (univariate analysis) of adjuvant therapies for PFS in *IDH* mutated, *IDH* wild-type, *TERT* promoter mutated and *TERT* promoter wild-type WHO grade II and III diffuse gliomas In *IDH* mutated WHO grade II and III diffuse gliomas, patients who received postoperative RT **A.** and CHT **B.** had significantly better PFS than those who did not. However, in *IDH* wild-type WHO grade II and III diffuse gliomas, PFS of patients who received postoperative RT **C.** or CHT **D.** did not differ significantly from PFS of those who did not. As for TERT promoter mutated and TERT promoter wild-type WHO grade II and III diffuse gliomas, patients who received postoperative RT **E, G.** and CHT **F, H.** had significantly better PFS than those who did not.

We further investigated the impact of genotoxic therapies on PFS in *IDH* mutated, *IDH* wild-type, *TERT* promoter mutated and *TERT* promoter wild-type WHO grade II and III diffuse gliomas. In univariate analysis, the prognostic significance of adjuvant genotoxic therapies (RT *p* < 0.001, CHT *p* < 0.001) on PFS was observed in the *IDH* mutated subgroup (*n* = 216), but not in the *IDH* wild-type subgroup (*n* = 79). Subsequent multivariate analysis demonstrated that WHO grade (*p* = 0.038), extent of resection (*p* < 0.001), RT (*p* = 0.001), and CHT (*p* = 0.026) were independent prognostic factors for PFS in *IDH* mutated WHO grade II and III diffuse gliomas (Fig. 2A-2B, Table 3, and Table S2). However, in *IDH* wild-type tumors, multivariate analysis showed that only WHO grade (*p* < 0.001) and extent of resection (*p* = 0.077) were of prognostic significance (Fig. 2C-2D, Table 3, and Table S2). As for *TERT* promoter mutated and wild-type WHO grade II and III gliomas, univariate analysis showed that RT and CHT were prognostic factors, significantly influencing PFS in the two subgroups (Fig. 2E-2H and Table S2). On subsequent multivariate analysis, however, only RT was an independent prognostic factor in *TERT* promoter mutated WHO grade II and III gliomas (Table 3).

**Table 3:** Multivariate analysis of clinicopathological factors for PFS in patients with *IDH* mutated (*n* = 215) and *IDH* wild-type (*n* = 77) WHO II and III diffuse gliomas

*IDH, TERT* promoter and 1p/19q codeletion status	Variables	PFS		OS	
		HR(95%CI)	*p*-value	HR(95%CI)	*p*-value
*IDH mut*	Age	1.011(0.990–1.031)	0.302	1.019(0.996–1.043)	0.105
	WHO grade (Grade II vs III)	1.720(1.030–2.873)	**0.038**	1.884(1.059–3.354)	**0.031**
	Complete resection (Yes vs. No)	2.399(1.559–3.691)	**<0.001**	1.960(1.212–3.169)	**0.006**
	RT (Yes vs. No)	2.345(1.409–3.904)	**0.001**	2.001(1.136–3.526)	**0.016**
	CHT (Yes vs. No)	1.646 (1.062–2.552)	**0.026**	1.295(0.781–2.149)	0.316
	1p/19q codeletion (Yes vs. No)	1.747(1.109–2.751)	**0.016**	2.142(1.270–3.612)	**0.004**
*IDH* wt	Age	1.028(1.005–1.052)	**0.017**	1.023(0.999–1.048)	0.056
	WHO grade (Grade II vs III)	4.030(1.978–8.208)	**<0.001**	4.113(1.910–8.858)	**<0.001**
	Complete resection (Yes vs. No)	2.398(0.908–6.330)	0.077	2.825(1.072–7.445)	**0.036**
	RT (Yes vs. No)	1.349(0.698–2.607)	0.373	1.367(0.701–2.667)	0.36
	CHT (Yes vs. No)	1.211(0.440–3.329)	0.711	0.903(0.339–2.400)	0.837
*TERT mut*	Age	1.057(1.027–1.088)	**<0.001**	1.085(1.047–1.125)	**<0.001**
	WHO grade (Grade II vs III)	2.214(1.150–4.262)	**0.017**	2.413(1.141–5.104)	**0.021**
	Complete resection (Yes vs. No)	2.387(1.307–4.358)	**0.005**	2.206(1.027–3.998)	**0.042**
	RT (Yes vs. No)	2.211(1.118–4.371)	**0.022**	2.292(1.055–4.978)	**0.036**
	CHT (Yes vs. No)	1.000(0.528–1.896)	1	0.577(0.271–1.230)	0.155
	1p/19q codeletion (Yes vs. No)	1.639(0.900–2.985)	0.107	2.209(1.028–4.004)	**0.041**
*TERT wt*	Age	1.022(1.005–1.039)	**0.011**	1.022(1.003–1.041)	**0.02**
	WHO grade (Grade II vs III)	2.87(1.755–4.692)	**<0.001**	3.282(1.930–5.579)	**<0.001**
	Complete resection (Yes vs. No)	1.967(1.241–3.118)	**0.004**	1.856(1.128–3.054)	**0.015**
	RT (Yes vs. No)	1.477(0.896–2.434)	0.126	1.551(0.917–2.622)	0.101
	CHT (Yes vs. No)	1.593 (0.991–2.561)	0.055	1.350(0.810–2.250)	0.249
	1p/19q codeletion (Yes vs. No)	2.085(0.898–4.844)	0.087	2.964(1.068–8.228)	**0.037**
1p/19q codeletion	Age	1.045(1.003–1.089)	**0.036**	1.066(1.010–1.125)	**0.021**
	WHO grade (Grade II vs III)	1.891(0.681–5.253)	0.221	2.448(0.777–7.708)	0.126
	Complete resection (Yes vs. No)	2.908(1.258–6.723)	**0.013**	3.167(1.207–8.310)	**0.019**
	RT (Yes vs. No)	2.404(0.953–6.066)	0.063	1.471(0.460–4.702)	0.515
	CHT (Yes vs. No)	1.526 (0.681–3.419)	0.304	0.798(0.277–2.302)	0.677
	1p/19q codeletion (Yes vs. No)	1.054(0.416–2.667)	0.912	0.810(0.267–2.460)	0.71

*p* values in bold were considered statistically significant

*IDH* mut: *IDH* mutant; *IDH* wt: *IDH* wild-type; HR: hazard ratio; CI: confidence interval; RT: radiation therapy; CHT: chemotherapy.

### Mutations in *TERT* promoter categorize *IDH* wild-type WHO grade II and III diffuse gliomas into two subgroups with different responses to adjuvant genotoxic therapies

**Figure 3:**
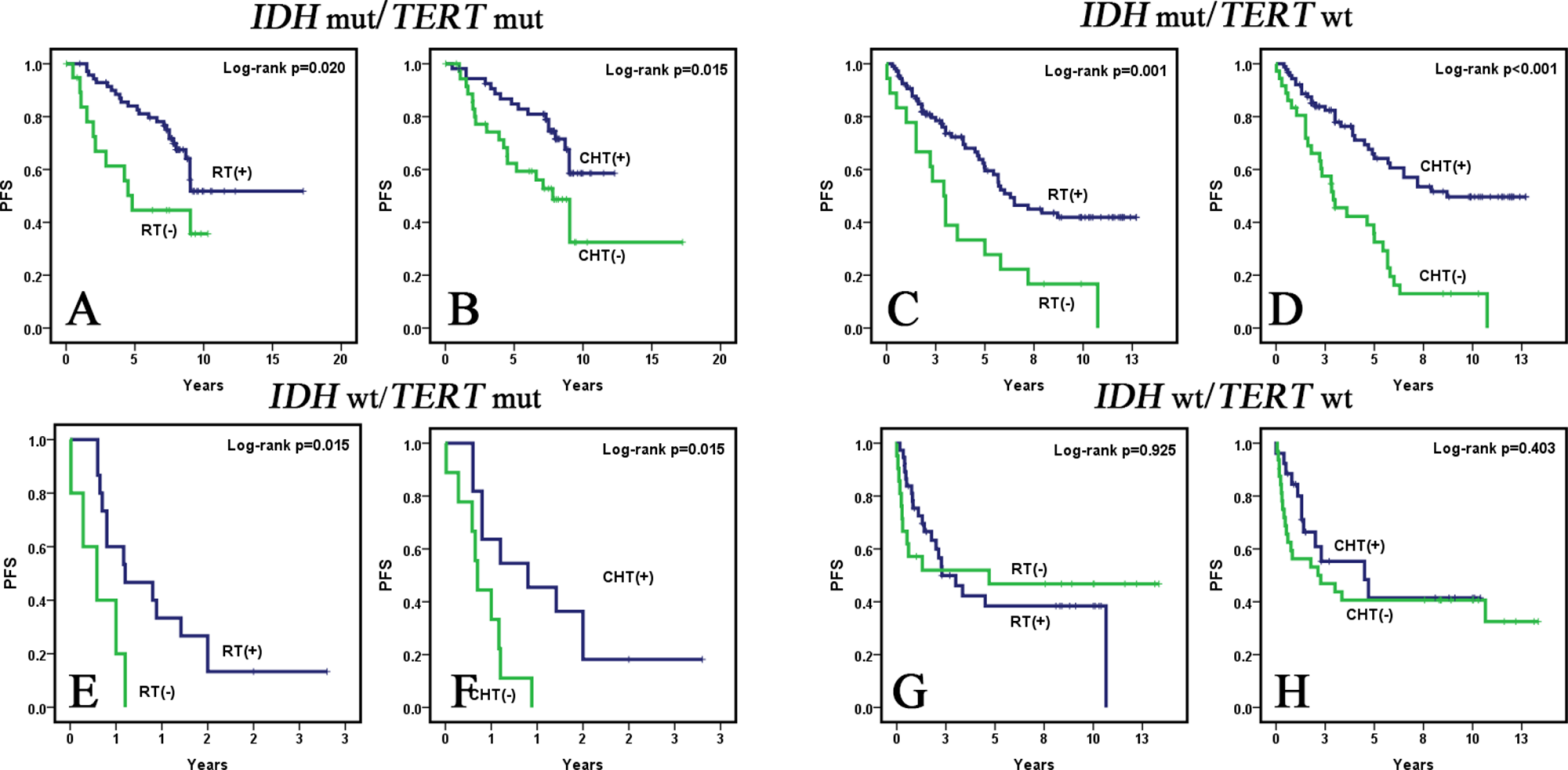
Kaplan-Meier survival curves (univariate analysis) of adjuvant therapies for PFS in subgroups of WHO grade II and III diffuse gliomas defined by *IDH* and *TERT* promoter mutations In *IDH* mut/*TERT* mut **A** and **B.** *IDH* mut/*TERT* wt **C** and **D.** and *IDH* wt/*TERT* mut tumors **E** and **F.**, patients who received post-operative RT and CHT had significantly better PFS than those who did not. However, in *IDH* wt/*TERT* wt tumors, PFS of patients who received postoperative RT **G.** and CHT **H.** did not differ significantly from PFS of those who did not.

We further combined *TERT* promoter and *IDH* mutations to stratify WHO grade II and III diffuse gliomas into four subgroups: *IDH* mut/*TERT* mut (*n* = 92), *IDH* mut/*TERT* wt (*n* = 125), *IDH* wt/*TERT* mut (*n* = 20) and *IDH* wt/*TERT* wt (*n* = 58). In *IDH* mutated WHO grade II and III diffuse gliomas (*IDH* mut/*TERT* mut and *IDH* mut/*TERT* wt). Genotoxic therapies significantly influenced PFS (Fig. 3A-3D, Table 4, and Table S3), with the exception that the administration of post-operative CHT did not reach statistical significance in multivariate analysis in the *IDH* mut/*TERT* mut subgroup (*p* = 0.369, Table 4). Upon univariate analysis of *IDH* wild-type WHO grade II and III diffuse gliomas, RT and CHT were significant parameters impacting the PFS in the *IDH* wt/*TERT* mut subgroup (RT *p* = 0.015, CHT *p* = 0.015) (Fig. 3E-3F, Table S3), but not in the *IDH* wt/*TERT* wt subgroup (RT *p* = 0.925, CHT *p* = 0.403) (Fig. 3G-3H, Table S3). Subsequent multivariate analysis confirmed that adjuvant therapies were not parameters significantly influencing PFS in the *IDH* wt/*TERT* wt subgroup (RT *p* = 0.598, CHT *p* = 0.741) (Table 4). Due to the limited sample size (*n* = 20) of the *IDH* wt/*TERT* mut subgroup, multivariate analysis was not performed.

**Table 4:** Multivariate analysis of clinicopathological factors for PFS in subgroups of WHO II and III diffuse gliomas as defined by *IDH* and *TERT* promoter mutations

*IDH*/1p/19q codeletion/*TERT* promoter status		PFS		OS	
	Variables	HR(95%CI)	*p*-value	HR(95%CI)	*p*-value
All patients	Age	1.028(1.015–1.042)	**<0.001**	1.031(1.016–1.047)	**<0.001**
	WHO grade (Grade II vs III)	2.549(1.733–3.749)	**<0.001**	2.802(1.838–4.271)	**<0.001**
	Complete resection (Yes vs. No)	2.026(1.413–2.906)	**<0.001**	1.848(1.241–2.751)	**0.002**
	RT (Yes vs. No)	1.671(0.968–2.503)	0.073	1.626(1.059–2.496)	**0.026**
	CHT (Yes vs. No)	1.337(0.917–1.951)	0.131	1.126(0.741–1.710)	0.578
	IDH mutation (Yes vs. No)	1.456(0.961–2.205)	0.076	1.803(1.176–2.763)	**0.007**
	1p/19q codeletion (Yes vs. No)	1.613(0.999–2.605)	0.051	1.881(1.088–3.252)	**0.024**
	*TERT* promoter mutation(Yes vs. No)	1.139(0.756–1.716)	0.533	1.158(0.746–1.796)	0.513
*IDH mut/TERT mut*	Age	1.041(1.005–1.079)	**0.024**	1.083(1.032–1.137)	**0.001**
	WHO grade (Grade II vs III)	2.016(0.860–4.728)	0.107	2.114(0.761–5.871)	0.151
	Complete resection (Yes vs. No)	2.628(1.273–5.423)	**0.009**	1.843(0.775–4.380)	0.167
	RT (Yes vs. No)	2.285(1.010–5.169)	**0.047**	1.976(0.725–5.386)	0.183
	CHT (Yes vs. No)	1.411(0.666–2.988)	0.369	0.690(0.250–1.902)	0.473
	1p/19q codeletion (Yes vs. No)	0.829(0.395–1.736)	0.618	0.878(0.353–2.183)	0.779
*IDH mut/TERT wt*	Age	1.008(0.981–1.036)	0.581	1.013(0.983–1.045)	0.393
	WHO grade (Grade II vs III)	1.838(0.923–3.659)	**0.083**	2.051(0.973–4.320)	0.059
	Complete resection (Yes vs. No)	2.718(1.522–4.854)	**0.001**	0.047(1.009–3.709)	**0.018**
	RT (Yes vs. No)	2.540(1.258–5.126)	**0.009**	1.917(0.933–3.937)	0.076
	CHT (Yes vs. No)	1.952(1.105–3.450)	**0.021**	1.617(0.855–3.059)	0.14
	1p/19q codeletion (Yes vs. No)	1.984(0.823–4.782)	0.127	2.849(0.986–8.229)	0.053
*IDH mut*/1p/19q codeletion	Age	1.045(1.003–1.089)	**0.036**	1.061(1.010–1.115)	**0.019**
	WHO grade (Grade II vs III)	1.891(0.681–5.253)	0.221	2.362(0.837–6.664)	0.104
	Complete resection (Yes vs. No)	2.908(1.258–6.723)	**0.013**	3.118(1.310–7.421)	**0.01**
	RT (Yes vs. No)	2.404(0.953–6.066)	0.063	1.069(0.387–2.951)	0.898
	CHT (Yes vs. No)	1.526(0.681–3.419)	0.304	0.852(0.334–2.174)	0.737
	*TERT* promoter mutation(Yes vs. No)	1.054(0.416–2.667)	0.912	0.890(0.349–2.267)	0.807
*IDH mut*/1p/19q intact	Age	1.011(0.981–1.042)	0.476	1.016(0.982–1.050)	0.373
	WHO grade (Grade II vs III)	2.474(1.072–5.709)	**0.034**	2.402(0.998–5.783)	0.051
	Complete resection (Yes vs. No)	3.050(1.513–6.145)	**0.002**	2.576(1.158–5.727)	**0.02**
	RT (Yes vs. No)	3.207(1.293–7.951)	**0.012**	3.187(1.250–8.125)	**0.015**
	CHT (Yes vs. No)	1.807(0.895–3.652)	**0.099**	1.830(0.832–4.025)	0.133
	*TERT* promoter mutation(Yes vs. No)	10.141(1.376–74.740)	**0.023**	2.782(0.753–9.166)	0.265
*IDH* wt/*TERT* wt	Age	1.014(0.986–1.042)	0.325	1.010(0.981–1.040)	0.509
	WHO grade (Grade II vs III)	5.114(2.075–12.602)	**<0.001**	5.460(2.083–14.317)	**0.001**
	Complete resection (Yes vs. No)	2.753(0.870–8.711)	0.085	3.115(0.966–10.041)	0.057
	RT (Yes vs. No)	1.243(0.553–2.791)	0.598	1.442(0.629–3.310)	0.387
	CHT (Yes vs. No)	1.230(0.361–4.191)	0.741	0.894(0.272–2.937)	0.854

*p* values in bold were considered statistically significant

*IDH* mut: *IDH* mutant; *IDH* wt: *IDH* wild-type; *TERT* mut: *TERT* promoter mutant; *TERT* wt: *TERT* promoter wild-type; HR: hazard ratio; CI: confidence interval; RT: radiation therapy; CHT: chemotherapy.

We further categorized *IDH* mutated WHO grade II and III gliomas into an *IDH* mutated, 1p/19q codeleted subgroup and an *IDH* mutated, 1p/19q intact subgroup. On multivariate analysis, only RT was an independent factor impacting the PFS of the *IDH* mutated, 1p/19q codeleted subgroup (*p* = 0.012, Table 4).

We also explored the roles of different chemotherapy strategies in the four subgroups defined by *IDH* and *TERT* promoter status. In the *IDH* mut/*TERT* mut and *IDH* mut/*TERT* wt subgroups, the PFS of patients receiving alkylating agents (MeCCNU, FCNU and ACNU) was significantly longer than those who did not receive chemotherapy (*IDH* mut/*TERT* mut *p* = 0.007, *IDH* mut/*TERT* wt *p* < 0.001), while the PFS of patients receiving TMZ as chemotherapy did not differ significantly from that of patients receiving alkylating agents (Figure S1, Table S4). As for the *IDH* wt/*TERT* mut and *IDH* wt/*TERT* wt subgroups, there was no significant difference between the PFS of patients who received TMZ, alkylating agents, and no chemotherapy (Figure S1, Table S4).

## DISCUSSION

Previously, we evaluated the frequency, distribution, and prognostic significance of *TERT* promoter mutations when combined with *IDH* mutations in WHO grade II to IV gliomas [19, 22]. The present study focused on the roles of *TERT* promoter mutations and *IDH* mutations in predicting responses to adjuvant genotoxic therapies. The results presented here confirmed that WHO grade II and III diffuse gliomas with *IDH* mutation are more sensitive to DNA-damaging therapies. Furthermore, tumors with *TERT* promoter mutations could further stratify *IDH* wild-type WHO grade II and III diffuse gliomas into two subsets with different responses to genotoxic therapies.

The discovery of *IDH* mutations is one of the most important findings in glioma genomics in recent years. The fact that *IDH* mutations confer a favorable prognosis for both PFS and OS in gliomas has been well established by numerous studies [3, 13–15, 23–26]. However, far fewer studies have addressed whether the superior PFS and OS of *IDH* mutated gliomas are due to less aggressive intrinsic tumor biology or due to improved sensitivity to genotoxic therapies. Dubbink et al observed no relationship between improved response to temozolomide chemotherapy and *IDH* mutations in progressive low-grade gliomas [13]. Likewise, a report from EORTC gave no indication that the presence of *IDH* mutations predicted improved response to procarbazine, 1-(2-chloroethyl)-3-cyclohexyl-L-nitrosourea, and vincristine (PCV) chemotherapy in WHO III anaplastic oligodendrogliomas and hypothesized that the favorable survival in *IDH* mutated gliomas was primarily due to a less aggressive biological behavior, rather than enhanced chemotherapeutic sensitivity [12]. Other studies, however, have drawn discrepant conclusions. Survival analysis of patients who never received post-operative adjuvant RT or CHT may be the closest approximation of the natural course of glioma and has been proposed to be the ideal model for studying the impact of *IDH* mutations on clinical outcomes [27]. Houillier et al. studied 171 patients without adjuvant therapies until first progression and demonstrated that spontaneous PFS did not differ significantly in patients with *IDH* mutated and wild-type low-grade gliomas [14]. Hartmann et al demonstrated similar results in an analysis of PFS in 89 patients with low-grade gliomas who received no additional genotoxic therapy after surgery [27]. More recently, in a study based on long-term follow-up data of RTOG trial 9402, *IDH* mutations were identified as a predictive biomarker that conferred survival benefit to patients with WHO III anaplastic oligodendrogliomas receiving PCV chemotherapy, but not in those without PCV chemotherapy [15]. In our cohort comprising patients who received (Group A) and did not receive (Group B) genotoxic therapies after surgery, we showed in univariate and multivariate analyses that *IDH* mutations were independent biomarkers significantly influencing PFS and OS in Group A but not in Group B (Table 2). Further analysis revealed that genotoxic therapies were independent clinical parameters impacting PFS in *IDH* mutated WHO grade II and III diffuse gliomas but not in *IDH* wild-type subgroups (Table 3). Moreover, multivariate analysis of the entire cohort including variables of genotoxic therapies (RT and CHT) and *IDH* mutation status revealed that both genotoxic therapies and *IDH* mutation status lost significance in the Cox regression model, demonstrating these variables are not independent prognostic factors and may in fact interact with one another (Table 4). These results raise the possibility that the favorable effects observed based on *IDH* mutation status and administration of adjuvant genotoxic therapy may be co-dependent. Thus, our study reinforces previous data, substantiating the hypothesis that *IDH* mutations confer improved survival due to enhanced chemotherapeutic sensitivity rather than from a more benign, intrinsic tumor biology.

*TERT* promoter mutations frequently occur across all types of gliomas, suggesting regulation of telomere elongation by telomerase may play an important role in the pathogenesis of gliomas [17, 18, 21]. Interest in the clinicopathological value of *TERT* promoter mutations has grown considerably in recent years. We are the first group to explore the potentially predictive role of *TERT* promoter mutations on response to genotoxic therapies in gliomas (Table 3). Notably, we found that *TERT* promoter mutations, in combination with *IDH* mutations, contribute to a survival benefit. We previously identified *TERT* promoter mutations as a favorable prognostication in tumors with *IDH* mutation, 1p/19q intact and an aggressive subset of tumors with wild-type *IDH* [22]. At that time, we also reported in a separate study utilizing a different cohort of WHO grade II to IV gliomas that *IDH* and *TERT* promoter mutations categorized four distinct subgroups in grade III and grade IV gliomas [19]. In this study, we sought to investigate the sensitivities to genotoxic therapies in subgroups with different *IDH* and *TERT* promoter mutations. We demonstrated that adjuvant therapies (RT and CHT) were significant clinical factors influencing PFS in three subgroups (*IDH* mut/*TERT* mut, *IDH* mut/*TERT* wt, *IDH* wt/*TERT* mut), but not in the *IDH* wt/*TERT* wt subgroup. These findings suggest that *TERT* promoter mutations may further stratify *IDH* wild-type gliomas, a subset previously considered to be less sensitive to adjuvant therapies than *IDH* mutated gliomas, into two subgroups with differential responses to genotoxic therapies. *IDH* wt/*TERT* mut gliomas were previously shown to exhibit a dismal prognosis and were most prevalent in primary GBM [19, 20]. Nonetheless, in our study, the *IDH* wt/*TERT* mut WHO grade II and III diffuse gliomas were more sensitive than *IDH* wt/*TERT* wt tumors to genotoxic therapies, raising the possibility that the intrinsic biological behaviors of this subtype might be more aggressive than others and that intensified treatment may be justified. Interestingly, we found that *IDH* wt/*TERT* wt WHO grade II and III diffuse gliomas did not respond to genotoxic therapies as well as other gliomas with either *IDH* mutations or *TERT* promoter mutations. While further investigations are needed, this finding supported previous work postulating that *IDH* wt/*TERT* wt WHO grade II and III diffuse gliomas represent a biologically and clinically distinct group [19]. Our data also suggested that the therapeutic efficacy of current genotoxic therapies in this subgroup was limited.

Recently, Suzuki et al categorized grade II and grade III gliomas into three distinct subtypes characterized by *IDH* mutations and 1p/19q codeletion [29]. Type I tumors were defined by the presence of both *IDH* mutations and 1p/19q codeletion. Type II tumors comprised of those tumors with *IDH* mutations and without 1p/19q codeletion, and type III tumors were *IDH* wild-type grade II and grade III gliomas. The three subsets were demonstrated to have distinctly genetic alterations and clinical behaviors. When we stratified our patient cohort into these three subsets, we found that only RT was an independent factor significantly influencing the PFS in type II (*IDH* mutated, 1p/19q intact) tumors (Table 4). Taking this into consideration with our findings that genotoxic therapies significantly prolonged PFS in *IDH* mut/*TERT* mut and *IDH* mut/*TERT* wt subgroups, we hypothesized that the *IDH* mutation may be a more important predictive marker than 1p/19q codeletion and *TERT* promoter mutations. Genotoxic therapies were independent prognostic factors in *IDH* mutated grade II and grade III gliomas but lost significance in *IDH* mutated subgroups divided by the status of 1p/19q codeletion and *TERT* promoter mutations. Furthermore, multivariate analysis on grade II and grade III gliomas with 1p/19q codeletion demonstrated that neither RT nor CHT was a significant, independent prognostic factor (Table 3). As such, *IDH* mutations may be predictive markers for genotoxic therapies in grade II and grade III gliomas as a whole, while 1p/19q codeletion status may only be predictive in certain histology types such as oligodendroglial and oligoastrocytic gliomas. Lastly, our study identified a potential role for *TERT* promoter mutations in classifying *IDH* wild-type tumors into two subsets with differential sensitivities to adjuvant genotoxic therapies as previously discussed.

There are several limitations and weaknesses in the present study. Although the total number of this patient cohort was relatively large, distribution among each subgroup was uneven and thus multivariate analysis could not be performed in one subgroup. Secondly, since the study was retrospective, protocols of adjuvant genotoxic therapies were not consistent. Therefore, the results of the present study should be used as a guide for future confirmation with standardized treatment protocols or clinical trials.

In conclusion, our study demonstrated that *IDH* mutated WHO grade II and III diffuse gliomas exhibit better PFS and OS than *IDH* wild-type subgroups when patients received genotoxic therapies post-operatively and that this survival benefit was lost when genotoxic therapies after surgery were absent. Our data also revealed that genotoxic therapies were independent favorable factors significantly influencing the outcome in *IDH* mutated WHO grade II and III diffuse gliomas but not in *IDH* wild-type tumors. Importantly, *TERT* promoter mutations stratified *IDH* wild-type WHO grade II and III diffuse gliomas into two subgroups with differential responses to adjuvant therapies. Overall, our study supports the role for *TERT* promoter mutations to complement *IDH* mutations in prognosticating WHO grade II and III diffuse gliomas in clinical practice.

## MATERIALS AND METHODS

### Patients and tissue samples

This study was approved by the Ethics Committee of Huashan Hospital, Fudan University and the New Territories East Cluster-Chinese University of Hong Kong Ethics Committee. A total of 295 patients pathologically diagnosed with WHO grade II and III diffuse gliomas in Huashan Hospital (Shanghai, China) and Prince of Wales Hospital (Hong Kong, China) between January 1990 and December 2013 were included in this study. The cohort of the study was partly overlapped with previous study [22]. Formalin-fixed paraffin embedded (FFPE) tissues, clinical data and follow-up data were analyzed. All cases were stained with hematoxylin & eosin (H&E) and centrally reviewed according to the 2007 World Health Organization (WHO) criteria by two senior neuropathologists (H.K.N. and Y.W.) [2]. In the series, there were 96 diffuse astrocytomas (WHO grade II; AII), 82 anaplastic astrocytomas (WHO grade III; AAIII), 29 oligodendrogliomas (WHO grade II; OII), 10 anaplastic oligodendrogliomas (WHO grade III; AOIII), 63 oligoastrocytomas (WHO grade II; OAII) and 15 anaplastic oligoastrocytomas (WHO grade III; AOAIII). Clinical and follow-up data were collected from medical charts, central radiological systems of the hospitals, out-patient clinics and telephone interviews. Progression-free survival (PFS) was measured from the date of pathological diagnosis to the date of initial tumor recurrence or progression (radiologically or pathologically). Radiological recurrence or progression was confirmed by magnetic resonance imaging (MRI) or computed tomography (CT). Pathological progression was confirmed by pathologists after second operation. Overall survival (OS) was measured from the date of pathological diagnosis to the date of death or last follow-up. The date of death was determined by cancellation of social ID.

### Analysis of molecular markers

Tumor DNA was extracted from FFPE tissue samples in all 295 cases in this cohort. Mutational hotspots of *IDH1* at codon 132 and *IDH2* at codon 172 were evaluated by direct sequencing as previously reported [23]. Mutational hotspots [chr5, 1, 295, 228 (C228T) and 1, 295, 250 (C250T)] in the *TERT* promoter region were evaluated by direct sequencing as previously reported [22]. Chromosome 1p/19q status was examined by fluorescence in situ hybridization as previously reported [22, 28].

### Statistical analysis

Survival curves were constructed using Kaplan-Meier methods. Differences in PFS and OS between subgroups of patients were analyzed by log-rank tests (univariate analysis). Suitable prognostic factors influencing the survival of WHO grade II and III diffuse gliomas were selected and subsequently put into Cox proportional hazards regression models to identify independent prognostic factors (multivariate analysis). Statistical significance was defined as a *p*-value of less than 0.05. Statistical analyses were performed using IBM SPSS Statistics 19 software (IBM Corp., Armonk, NY, USA).

## SUPPLEMENTARY FIGURES AND TABLES



## References

[1] Wen PY, Kesari S (2008). Malignant gliomas in adults. N Engl J Med.

[2] Louis DN, Ohgaki H, Wiestler OD, Cavenee WK, Burger PC, Jouvet A, Scheithauer BW, Kleihues P (2007). The 2007 WHO classification of tumours of the central nervous system. Acta Neuropathol.

[3] Sabha N, Knobbe CB, Maganti M, Al Omar S, Bernstein M, Cairns R, Çako B, von Deimling A, Capper D, Mak TW, Kiehl TR, Carvalho P, Garrett E (2014). Analysis of IDH mutation, 1p/19q deletion, and PTEN loss delineates prognosis in clinical low-grade diffuse gliomas. Neuro Oncol.

[4] Forst DA, Nahed BV, Loeffler JS, Batchelor TT (2014). Low-grade gliomas. Oncologist.

[5] Cavaliere R, Lopes MB, Schiff D (2005). Low-grade gliomas: an update on pathology and therapy. Lancet Neurol.

[6] Smith JS, Perry A, Borell TJ, Lee HK, O'Fallon J, Hosek SM, Kimmel D, Yates A, Burger PC, Scheithauer BW, Jenkins RB (2000). Alterations of chromosome arms 1p and 19q as predictors of survival in oligodendrogliomas, astrocytomas, and mixed oligoastrocytomas. J Clin Oncol.

[7] van den Bent MJ, Brandes AA, Taphoorn MJ, Kros JM, Kouwenhoven MC, Delattre JY, Bernsen HJ, Frenay M, Tijssen CC, Grisold W, Sipos L, Enting RH, French PJ (2013). Adjuvant procarbazine, lomustine, and vincristine chemotherapy in newly diagnosed anaplastic oligodendroglioma: long-term follow-up of EORTC brain tumor group study 26951. J Clin Oncol.

[8] Cairncross G, Wang M, Shaw E, Jenkins R, Brachman D, Buckner J, Fink K, Souhami L, Laperriere N, Curran W, Mehta M (2013). Phase III trial of chemoradiotherapy for anaplastic oligodendroglioma: long-term results of RTOG 9402. J Clin Oncol.

[9] Yan H, Parsons DW, Jin G, McLendon R, Rasheed BA, Yuan W, Kos I, Batinic-Haberle I, Jones S, Riggins GJ, Friedman H, Friedman A, Reardon D (2009). IDH1 and IDH2 mutations in gliomas. N Engl J Med.

[10] Ichimura K, Pearson DM, Kocialkowski S, Bäcklund LM, Chan R, Jones DT, Collins VP (2009). IDH1 mutations are present in the majority of common adult gliomas but rare in primary glioblastomas. Neuro Oncol.

[11] Sanson M, Marie Y, Paris S, Idbaih A, Laffaire J, Ducray F, El Hallani S, Boisselier B, Mokhtari K, Hoang-Xuan K, Delattre JY (2009). Isocitrate dehydrogenase 1 codon 132 mutation is an important prognostic biomarker in gliomas. J Clin Oncol.

[12] van den Bent MJ, Dubbink HJ, Marie Y, Brandes AA, Taphoorn MJ, Wesseling P, Frenay M, Tijssen CC, Lacombe D, Idbaih A, van Marion R, Kros JM, Dinjens WN (2010). IDH1 and IDH2 mutations are prognostic but not predictive for outcome in anaplastic oligodendroglial tumors: a report of the European Organization for Research and Treatment of Cancer Brain Tumor Group. Clin Cancer Res.

[13] Dubbink HJ, Taal W, van Marion R, Kros JM, van Heuvel I, Bromberg JE, Zonnenberg BA, Zonnenberg CB, Postma TJ, Gijtenbeek JM, Boogerd W, Groenendijk FH, Smitt PA (2009). IDH1 mutations in low-grade astrocytomas predict survival but not response to temozolomide. Neurology.

[14] Houillier C, Wang X, Kaloshi G, Mokhtari K, Guillevin R, Laffaire J, Paris S, Boisselier B, Idbaih A, Laigle-Donadey F, Hoang-Xuan K, Sanson M, Delattre JY (2010). IDH1 or IDH2 mutations predict longer survival and response to temozolomide in low-grade gliomas. Neurology.

[15] Cairncross JG, Wang M, Jenkins RB, Shaw EG, Giannini C, Brachman DG, Buckner JC, Fink KL, Souhami L, Laperriere NJ, Huse JT, Mehta MP, Curran WJ (2014). Benefit from procarbazine, lomustine, and vincristine in oligodendroglial tumors is associated with mutation of IDH. J Clin Oncol.

[16] Huang FW, Hodis E, Xu MJ, Kryukov GV, Chin L, Garraway LA (2013). Highly recurrent TERT promoter mutations in human melanoma. Science.

[17] Killela PJ, Reitman ZJ, Jiao Y, Bettegowda C, Agrawal N, Diaz LA, Friedman AH, Friedman H, Gallia GL, Giovanella BC, Grollman AP, He TC, He Y (2013). TERT promoter mutations occur frequently in gliomas and a subset of tumors derived from cells with low rates of self-renewal. Proc Natl Acad Sci U S A.

[18] Koelsche C, Sahm F, Capper D, Reuss D, Sturm D, Jones DT, Kool M, Northcott PA, Wiestler B, Böhmer K, Meyer J, Mawrin C, Hartmann C (2013). Distribution of TERT promoter mutations in pediatric and adult tumors of the nervous system. Acta Neuropathol.

[19] Killela PJ, Pirozzi CJ, Healy P, Reitman ZJ, Lipp E, Rasheed BA, Yang R, Diplas BH, Wang Z, Greer PK, Zhu H, Wang CY, Carpenter AB (2014). Mutations in IDH1, IDH2, and in the TERT promoter define clinically distinct subgroups of adult malignant gliomas. Oncotarget.

[20] Nonoguchi N, Ohta T, Oh JE, Kim YH, Kleihues P, Ohgaki H (2013). TERT promoter mutations in primary and secondary glioblastomas. Acta Neuropathol.

[21] Arita H, Narita Y, Fukushima S, Tateishi K, Matsushita Y, Yoshida A, Miyakita Y, Ohno M, Collins VP, Kawahara N, Shibui S, Ichimura K (2013). Upregulating mutations in the TERT promoter commonly occur in adult malignant gliomas and are strongly associated with total 1p19q loss. Acta Neuropathol.

[22] Chan AK, Yao Y, Zhang Z, Chung NY, Liu JS, Li KK, Shi Z, Chan DT, Poon WS, Zhou L, Ng HK (2015). TERT promoter mutations contribute to subset prognostication of WHO grade II and III diffuse gliomas. Mod Pathol.

[23] Yao Y, Chan AK, Qin ZY, Chen LC, Zhang X, Pang JC, Li HM, Wang Y, Mao Y, Ng HK, Zhou LF (2013). Mutation analysis of IDH1 in paired gliomas revealed IDH1 mutation was not associated with malignant progression but predicted longer survival. PLoS One.

[24] Hartmann C, Hentschel B, Wick W, Capper D, Felsberg J, Simon M, Westphal M, Schackert G, Meyermann R, Pietsch T, Reifenberger G, Weller M, Loeffler M (2010). Patients with IDH1 wild-type anaplastic astrocytomas exhibit worse prognosis than IDH1-mutated glioblastomas, and IDH1 mutation status accounts for the unfavorable prognostic effect of higher age: implications for classification of gliomas. Acta Neuropathol.

[25] Hartmann C, Meyer J, Balss J, Capper D, Mueller W, Christians A, Felsberg J, Wolter M, Mawrin C, Wick W, Weller M, Herold-Mende C, Unterberg A (2009). Type and frequency of IDH1 and IDH2 mutations are related to astrocytic and oligodendroglial differentiation and age: a study of 1, 010 diffuse gliomas. Acta Neuropathol.

[26] Weller M, Felsberg J, Hartmann C, Berger H, Steinbach JP, Schramm J, Westphal M, Schackert G, Simon M, Tonn JC, Heese O, Krex D, Nikkhah G (2009). Molecular predictors of progression-free and overall survival in patients with newly diagnosed glioblastoma: a prospective translational study of the German Glioma Network. J Clin Oncol.

[27] Hartmann C, Hentschel B, Tatagiba M, Schramm J, Schnell O, Seidel C, Stein R, Reifenberger G, Pietsch T, von Deimling A, Loeffler M (2011). Molecular markers in low-grade gliomas: predictive or prognostic?. Clin Cancer Res.

[28] Dong Z, Pang JS, Ng MH, Poon WS, Zhou L, Ng HK (2004). Identification of two contiguous minimally deleted regions on chromosome 1p36.31-p36.32 in oligodendroglial tumours. Br J Cancer.

[29] Suzuki H, Aoki K, Chiba K (2015). Mutational landscape and clonal architecture in grade II and III gliomas. Nat Genet.

