# Supplementary material for: TERT promoter mutations contribute to IDH mutations in predicting differential responses to adjuvant therapies in WHO grade II and III diffuse gliomas
Source: Oncotarget. 2015 Jul 9;6(28):24871–83. doi: 10.18632/oncotarget.4549 (PMC4694799; doi:10.18632/oncotarget.4549)
Supplement: Supplementary file 1 [file oncotarget-06-24871-s001.pdf]

SUPPLEMENTARY FIGURES AND TABLES

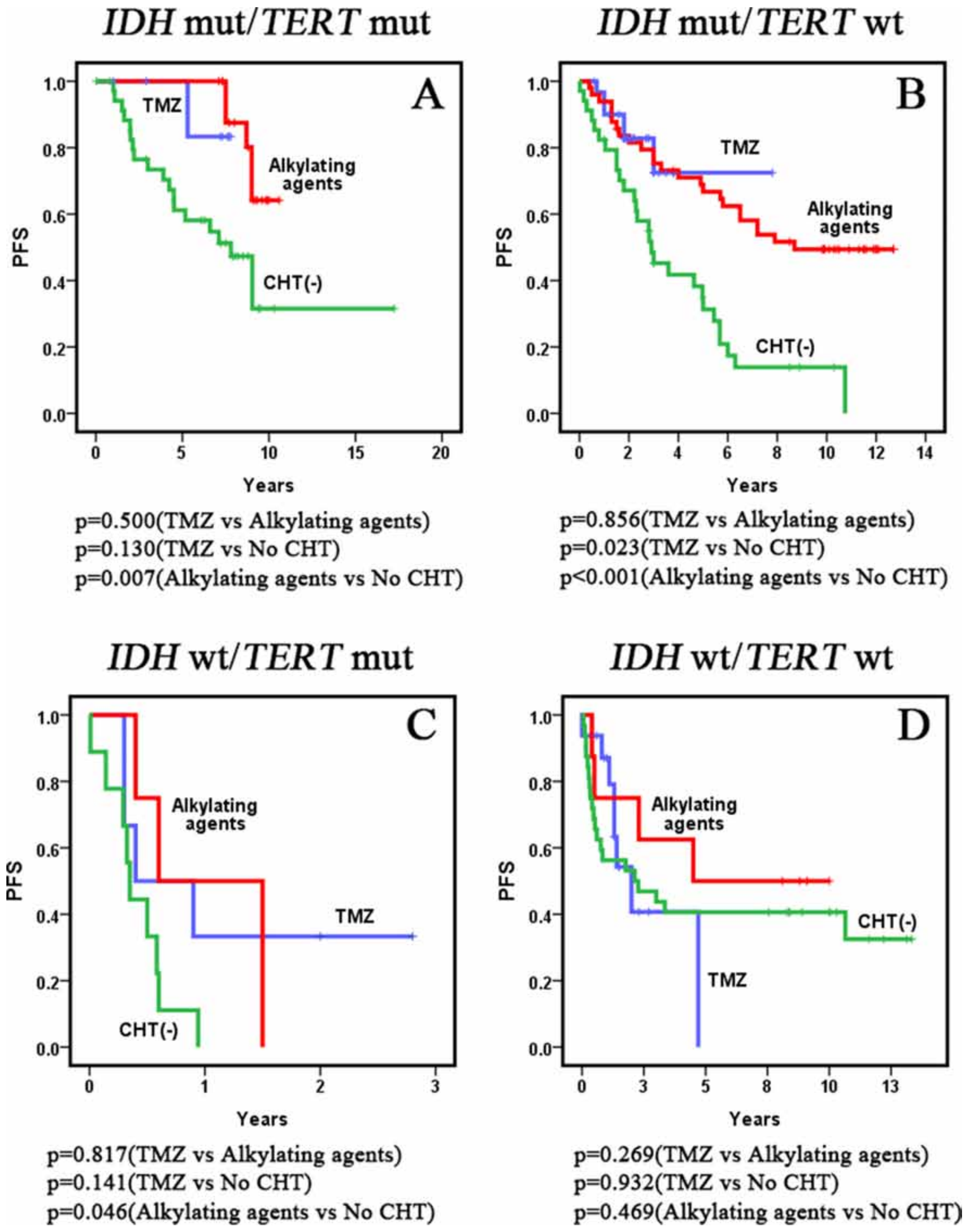

Supplementary Figure S1: Kaplan-Meier survival curves (univariate analysis) of different chemotherapy schemes for PFS in subgroups of WHO grade II and III diffuse gliomas defined by *IDH* and *TERT* promoter mutations. In *IDH* mut/*TERT* mut **A.** *IDH* mut/*TERT* wt **B.** *IDH* wt/*TERT* mut **C.** and *IDH* wt/*TERT* wt **D.** subgroups, the PFS of patients who received TMZ (blue) did not differ significantly from the PFS of those who received other alkylating agents (red).

Supplementary Table S1. Univariate analysis of pathological factors for PFS and OS in patients with WHO grade II and III diffuse gliomas who received adjuvant therapies (Group A, *n* = 246) and who did not (Group B, *n* = 49) after first surgery

Adjuvant therapies	Molecular markers	*N*	Median PFS (years)	*p*-value	Median OS (years)	*p*-value
RT and/or CHT		246				
	IDH					
	IDH mut	189	9.0	**<0.001**	NR	**<0.001**
	IDH wt	57	1.8		2.6	
	TERT promoter					
	TERT mut	93	9.0	0.072	NR	**0.046**
	TERT wt	153	5.7		7.8	
No RT nor CHT\		49				
	IDH					
	IDH mut	27	2.9	0.702	9.7	0.497
	IDH wt	22	0.5		0.6	
	TERT promoter					
	TERT mut	19	2.0	0.706	9.7	0.851
	TERT wt	30	2.3		3.6	

p values in bold were considered statistically significant
IDH mut : *IDH* mutant; *IDH* wt: *IDH* wild-type; *TERT* mut: *TERT* promoter mutant;
TERT wt *TERT* promoter wild-type; RT: radiation therapy; CHT: chemotherapy;
PFS: progression-free survival; OS: overall-survival; NR: not reached

Supplementary Table S2. Univariate analysis of adjuvant therapies for PFS and OS in patients with *IDH* mutated WHO grade II and III diffuse gliomas (*n* = 216), *IDH* wild-type lower grade gliomas (*n* = 79), TERT promoter mutated WHO grade II and III diffuse gliomas (*n* = 112) and TERT promoter wild-type WHO grade II and III diffuse gliomas (*n* = 183)

***IDH* or *TERT* status**	**Adjuvant therapies**	***N***	**Median PFS (years)**	***p*-value**	**Median OS (years)**	***p*-value**
IDH mut		216				
	RT					
	Yes	178	9	**<0.001**	NR	**0.004**
	No	38	3.6		7.8	
	CHT					
	Yes	142	NR	**<0.001**	NR	**0.001**
	No	74	5		8.8	
IDH wt		79				
	RT					
	Yes	53	1.8	0.774	2.5	0.844
	No	26	0.6		1.8	
	CHT					
	Yes	38	2	0.273	2.6	0.480
	No	41	0.8		1.8	
TERT mut		112				
	RT					
	Yes	87	9	**0.016**	NR	0.080
	No	25	2.9		9.7	
	CHT					
	Yes	65	NR	**0.010**	NR	0.094
	No	47	4.5		9.7	
TERT wt		183				
	RT					
	Yes	144	5.7	**0.034**	9.1	**0.032**
	No	39	3		5	
	CHT					
	Yes	115	7.9	**<0.001**	11.9	**0.003**
	No	68	2.8		4.8	

p values in bold were considered statistically significant
IDH mut : *IDH* mutant; *IDH* wt: *IDH* wild-type; RT: radiation therapy; CHT: chemotherapy; PFS: progression-free survival; OS: overall-survival

Supplementary Table S3. Univariate analysis of adjuvant therapies for PFS and OS in subgroups of WHO grade II and III diffuse gliomas defined by *IDH* and *TERT* promoter mutations

***IDH*/*TERT* status**	**Adjuvant therapies**	***N***	**Median PFS (years)**	***p*-value**	**Median OS (years)**	***p*-value**
IDH mut/*TERT* mut		91				
	RT					
	Yes	72	NR	**0.020**	NR	0.160
	No	20	4.8		11.3	
	CHT					
	Yes	54	NR	**0.015**	NR	0.219
	No	38	7.8		11.3	
IDH mut/*TERT* wt		125				
	RT					
	Yes	107	6.3	**0.001**	11.9	**0.013**
	No	18	2.9		4.7	
	CHT					
	Yes	89	8.7	**<0.001**	11.9	**0.001**
	No	36	2.9		5.3	
IDH wt/*TERT* mut		20				
	RT					
	Yes	15	0.6	**0.015**	1.8	**<0.001**
	No	5	0.3		0.4	
	CHT					
	Yes	11	0.9	**0.015**	2.5	**<0.001**
	No	9	0.4		0.5	
IDH wt/*TERT* wt		58				
	RT					
	Yes	37	2.3	0.925	4.8	0.769
	No	21	4.7		5.0	
	CHT					
	Yes	26	4.5	0.403	5	0.578
	No	32	2.1		3.9	

p values in bold were considered statistically significant
IDH mut : *IDH* mutant; *IDH* wt: *IDH* wild-type; *TERT* mut: *TERT* promoter mutant;
TERT wt: *TERT* promoter wild-type; RT: radiation therapy; CHT: chemotherapy PFS: progression-free survival; OS: overall-survival; NR: not reached

Supplementary Table S4. Univariate analysis of different strategies in chemotherapy for PFS and OS in subgroups of WHO grade II and III diffuse gliomas defined by *IDH* and *TERT* promoter mutations

***IDH/TERT* status**	**CHT protocol**	***N*[a]**	**Median PFS (years)**	***p*-value**
IDH mut/*TERT* mut		63		
	TMZ	8	NR	0.500[b] (TMZ vs Alkylating agents)
	Alkylating agents	19	NR	0.130[b] (TMZ vs No CHT)
	No CHT	36	7.8	**0.007[b]** (Alkylating agents vs No CHT)
IDH mut/*TERT* wt		114		
	TMZ	31	NR	0.856[b] (TMZ vs Alkylating agents)
	Alkylating agents	49	8.7	0.023[b] (TMZ vs No CHT)
	No CHT	34	2.9	**<0.001[b]** (Alkylating agents vs No CHT)
IDH wt/*TERT* mut		19		
	TMZ	6	0.4	0.817[b] (TMZ vs Alkylating agents)
	Alkylating agents	4	0.6	0.141[b] (TMZ vs No CHT)
	No CHT	9	0.3	0.046[b] (Alkylating agents vs No CHT)
IDH wt/*TERT* wt		56		
	TMZ	16	2	0.269[b] (TMZ vs Alkylating agents)
	Alkylating agents	8	4.5	0.932[b] (TMZ vs No CHT)
	No CHT	32	2.1	0.469[b] (Alkylating agents vs No CHT)

p values in bold were considered statistically significant

[a]Those cases with unavailable chemotherapy protocols were excluded

[b]To correct for multiple comparisons, a Bonferroni adjusted *p* value of 0.05/3 (3 = number of times of comparisons) = 0.017 was adopted as the significance threshold
